# MIPD: Molecules, Imagings, and Clinical Phenotype Integrated Database

**DOI:** 10.1093/database/baaf029

**Published:** 2025-04-21

**Authors:** Jiaojiao Zhao, Min Wu, Meihua Wan, Xue Li, Jie Li, Qin Liu, Minghao Xiong, Mengjie Tu, Jun Zhou, Shilin Li, Jie Zhang, Jiangping Fu, Yin Zhang, Chungang Zhao, Litong Qin, Xue Yang, Hong Zhao, Yan Zhang, Fanxin Zeng

**Affiliations:** Department of Clinical Research Center, Sichuan Clinical Research Center for Medical Imaging, Dazhou Key Laboratory for Precision Cancer Therapy, Dazhou Key Laboratory for Artificial Intelligence and Medical Imaging, Dazhou Central Hospital, 56 Nan Yue Miao Street, Dazhou 635000, China; Huaxi MR Research Center, Department of Radiology, West China Hospital of Sichuan University, 37 Guo Xue Xiang, Chengdu 610041, China; West China Center of Excellence for Pancreatitis, Institute of Integrated Traditional Chinese and Western Medicine, West China Hospital of Sichuan University, 37 Guo Xue Xiang, Chengdu 610041, China; Department of Clinical Research Center, Sichuan Clinical Research Center for Medical Imaging, Dazhou Key Laboratory for Precision Cancer Therapy, Dazhou Key Laboratory for Artificial Intelligence and Medical Imaging, Dazhou Central Hospital, 56 Nan Yue Miao Street, Dazhou 635000, China; Department of Clinical Research Center, Sichuan Clinical Research Center for Medical Imaging, Dazhou Key Laboratory for Precision Cancer Therapy, Dazhou Key Laboratory for Artificial Intelligence and Medical Imaging, Dazhou Central Hospital, 56 Nan Yue Miao Street, Dazhou 635000, China; Department of Clinical Research Center, Sichuan Clinical Research Center for Medical Imaging, Dazhou Key Laboratory for Precision Cancer Therapy, Dazhou Key Laboratory for Artificial Intelligence and Medical Imaging, Dazhou Central Hospital, 56 Nan Yue Miao Street, Dazhou 635000, China; Department of Clinical Research Center, Sichuan Clinical Research Center for Medical Imaging, Dazhou Key Laboratory for Precision Cancer Therapy, Dazhou Key Laboratory for Artificial Intelligence and Medical Imaging, Dazhou Central Hospital, 56 Nan Yue Miao Street, Dazhou 635000, China; Department of Clinical Research Center, Sichuan Clinical Research Center for Medical Imaging, Dazhou Key Laboratory for Precision Cancer Therapy, Dazhou Key Laboratory for Artificial Intelligence and Medical Imaging, Dazhou Central Hospital, 56 Nan Yue Miao Street, Dazhou 635000, China; Department of Clinical Research Center, Sichuan Clinical Research Center for Medical Imaging, Dazhou Key Laboratory for Precision Cancer Therapy, Dazhou Key Laboratory for Artificial Intelligence and Medical Imaging, Dazhou Central Hospital, 56 Nan Yue Miao Street, Dazhou 635000, China; Department of Clinical Research Center, Sichuan Clinical Research Center for Medical Imaging, Dazhou Key Laboratory for Precision Cancer Therapy, Dazhou Key Laboratory for Artificial Intelligence and Medical Imaging, Dazhou Central Hospital, 56 Nan Yue Miao Street, Dazhou 635000, China; Department of Clinical Research Center, Sichuan Clinical Research Center for Medical Imaging, Dazhou Key Laboratory for Precision Cancer Therapy, Dazhou Key Laboratory for Artificial Intelligence and Medical Imaging, Dazhou Central Hospital, 56 Nan Yue Miao Street, Dazhou 635000, China; Department of Oncology, Dazhou Central Hospital, 56 Nan Yue Miao Street, Dazhou 635000, China; Department of Oncology, Dazhou Central Hospital, 56 Nan Yue Miao Street, Dazhou 635000, China; Department of Radiology, Dazhou Central Hospital, 56 Nan Yue Miao Street, Dazhou 635000, China; Department of Clinical Research Center, Sichuan Clinical Research Center for Medical Imaging, Dazhou Key Laboratory for Precision Cancer Therapy, Dazhou Key Laboratory for Artificial Intelligence and Medical Imaging, Dazhou Central Hospital, 56 Nan Yue Miao Street, Dazhou 635000, China; Department of Clinical Research Center, Sichuan Clinical Research Center for Medical Imaging, Dazhou Key Laboratory for Precision Cancer Therapy, Dazhou Key Laboratory for Artificial Intelligence and Medical Imaging, Dazhou Central Hospital, 56 Nan Yue Miao Street, Dazhou 635000, China; Department of Hepatobiliary Surgery, State Key Laboratory of Molecular Oncology, National Cancer Center/National Clinical Research Center for Cancer/Cancer Hospital, Chinese Academy of Medical Sciences and Peking Union Medical College, 9 Dong Dan San Tiao, Beijing 100000, China; Lung Cancer Center, West China Hospital of Sichuan University, 37 Guo Xue Xiang, Chengdu 610041, China; Department of Clinical Research Center, Sichuan Clinical Research Center for Medical Imaging, Dazhou Key Laboratory for Precision Cancer Therapy, Dazhou Key Laboratory for Artificial Intelligence and Medical Imaging, Dazhou Central Hospital, 56 Nan Yue Miao Street, Dazhou 635000, China

## Abstract

Due to tumor heterogeneity, a subset of patients fails to benefit from current treatment strategies. However, an integrated analysis of imaging features, genetic molecules, and clinical phenotypes can characterize tumor heterogeneity, enabling the development of more personalized treatment approaches. Despite its potential, cross-modal databases remain underexplored. To address this gap, we established a comprehensive database encompassing 9965 genes, 5449 proteins, 1121 metabolites, 283 pathways, 854 imaging features, and 73 clinical factors from colorectal cancer patients. This database identifies significantly distinct molecules and imaging features associated with clinical phenotypes and provides survival analysis based on these features. Additionally, it offers genetic molecule annotations, comparative expression levels between tumor and normal tissues, imaging features linked to genetic molecules, and imaging-based models for predicting gene expression levels. Furthermore, the database highlights correlations between genetic molecules, clinical factors, and imaging features.

In summary, we present MIPD (Molecules, Imaging, and Clinical Phenotype Correlation Database), a user-friendly, interactive, and specialized platform accessible at http://corgenerf.com. MIPD facilitates the interpretability of cross-modal data by providing query, browse, search, visualization, and download functionalities, thereby offering a valuable resource for advancing precision medicine in colorectal cancer.

**Database URL**: http://corgenerf.

## Introduction

Colorectal cancer (CRC) is one of the leading causes of cancer-related mortality, and its disease burden continues to rise alongside increasing incidence rates [1, 2]. Genetic molecules play a pivotal role in the tumorigenesis and progression of CRC [3, 4], significantly contributing to early diagnosis, screening, prognosis prediction, molecular classification, and therapeutic decision-making [5, 6]. Furthermore, a large amount of imaging features can be extracted from medical images following the radiomics emerging [7, 8]. These features have been shown to correlate significantly with molecular subtypes, disease statuses, and treatment responses [9–11]. Alterations in genetic molecules can lead to changes at the tissue level, which may manifest as distinct outcomes detectable through medical imaging and laboratory tests. Given the close interrelationship between genetic molecules, imaging features, and clinical phenotypes, the integrated analysis of these multi-omics data holds great promise for optimizing treatment strategies and advancing precision medicine in CRC.

Currently, numerous databases have been established to support gene expression analysis, gene-related correlations, and access to medical imaging data. An influential database in this regard is The Cancer Genome Atlas (TCGA), which contains a wide range of genomic information and gene expression data [12]. To further investigate gene correlations, the cBioPortal platform offers intuitive visualization tools, while the Gene Expression Profiling Interactive Analysis (GEPIA) tool allows for comprehensive gene expression analysis [13, 14]. Additionally, several specialized databases have been developed to explore diverse types of gene correlations, including gene–gene interactions, gene–protein associations, gene–viral infection relationships, and networks for gene co-expression and metabolite co-accumulation [15–19]. Furthermore, The Cancer Imaging Archive (TCIA) serves as a critical resource by providing public access to a wide range of cancer-related medical images. Despite these advancements, most existing databases primarily focus on providing clinical factors, sequencing data, medical images, gene expression analysis, and gene-related correlations, leaving significant gaps in the integration of multi-modal data.

In previous studies, we have developed imaging-based models to predict gene expression levels and further elucidated their utility in predicting lymph node metastasis by investigating the biological functions of genes and pathways associated with imaging features in CRC [20, 21]. Moreover, numerous studies have explored the underlying biological mechanisms through which imaging features can inform clinical decision-making [22]. These imaging features, which capture intratumor heterogeneity and correlate with prognosis, are closely linked to gene expression patterns and pathways involved in immune response, tumor proliferation, and treatment response across various cancer types [23–26]. Additionally, imaging features provide valuable insights into genomics, enabling the prediction of gene expression levels and the mutational status of key genes and pathways [20, 25, 27].

However, research on the integration of diverse models and databases remains limited. Therefore, there is a critical need to develop a comprehensive database that investigates the correlations among genetic molecules, genotype pathways, functional types, imaging phenotypes, and clinical phenotypes. To address this gap, we have developed a user-friendly, interactive, and large-scale database named the Molecules, Imaging, and Clinical Phenotype Correlation Database (MIPD; http://corgenerf.com/). In conclusion, MIPD offers a range of functionalities, including query, browsing, searching, visualization, and data download, enabling researchers to explore and interpret cross-modal data effectively.

## Materials and methods

### Data collection

This study was approved by the ethics standards of the Clinical Research Ethics Committee of Dazhou Central Hospital (IRB00000003-17003), in accordance with the Declaration of Helsinki and informed consent. A total of 942 patients with pathologically proven CRC in Dazhou Central Hospital from January 2014 to October 2019 were included in this study.

The study incorporated six datasets, including imaging, RNA, pathway, protein, metabolite, and clinical datasets (Figure S1). The imaging dataset included 591 CRC patients with high-quality computer tomography (CT) Digital Imaging and Communications in Medicine (DICOM) data of the portal vein (slice thickness 1.0 mm). The RNA dataset included 142 CRC patients with RNA sequencing (RNA-seq) data. The transcriptomic data were reprocessed into transcripts per million (TPM) data for subsequent correlation analysis. Genes were further filtered by matching with four public databases: the Network of Cancer Genes & Healthy Drivers (NCG7.0) [28], oncogene database [29], Tumor Suppressor Gene Database (TSGene) [30, 31], Tissue-specific Gene DataBase [32]. Healthy drivers from NCG7.0 were excluded. Total of 2297 genes were selected from the original 58 435 genes based on matching with the aforementioned databases and the medium TPM value > 0. These genes were subsequently used for enrichment analysis, leading to the identification of 283 enriched pathways. The pathway dataset included these 283 pathways, which are known to play critical roles in cancer progression and metastasis.

The protein dataset included 31 CRC patients with 5620 proteins, filtered based on the following criteria: relative expression values >1 and selection of duplicate proteins based on higher abundance values. The metabolite dataset included 56 CRC patients with 1165 metabolites, filtered based on median relative abundance values > 1 and selection of duplicate metabolites based on higher abundance values. The clinical dataset included 159 CRC patients with 73 clinical factors after excluding patients without survival data. Clinical data were obtained from the medical record system, and missing values were imputed using the mean values of the respective features (Figure S1).

### Imaging feature extraction

All 591 enrolled CRC patients underwent preoperative contrast-enhanced abdominal CT scans using a SOMATOM Definition AS 64-slice CT scanner (SIEMENS, Germany). The DICOM data were retrieved from the Picture Archiving and Communication System (PACS), and tumor regions were manually segmented by two radiologists using 3D Slicer software (version 4.10.2). A total of 854 imaging features were extracted using the SlicerImaging plug-in, including first-order statistics, shape-related features, gray-level co-occurrence matrix, gray-level dependence matrix, gray-level run length matrix, gray-level size zone matrix, and neighboring gray-tone dependence matrix features, both with original and wavelet transformations. Detailed descriptions of these imaging features were annotated based on a previously published study [33].

### Sequencing analysis and pathway enriched

Tumor tissues and adjacent normal tissues were collected during surgery and immediately stored in liquid nitrogen. RNA extraction, sequencing, and analysis were performed following previously established protocols [20]. A total of 58 435 genes were identified after sequencing using the Illumina HiSeq PE150 platform. From these, 13 949 genes were selected based on a median TPM value > 1. Pathway-enrichment analysis was conducted using the “GSVA” package with reference to 833 signaling pathways from the KEGG, REACTOME, and BIOCARTA databases, yielding enrichment pathway scores. Gene descriptions were annotated using the National Center for Biotechnology Information (NCBI) database and the “clusterProfiler” package.

Tissue samples were individually ground in liquid nitrogen and lysed using PASP lysis buffer. The lysate was centrifuged, and the supernatant was reduced with dithiothreitol, followed by alkylation with iodoacetamide. The samples were then thoroughly mixed with acetone, centrifuged, and the resulting precipitate was collected. After washing with cold acetone, the pellet was dissolved in dissolution buffer. Protein quality was assessed using a Bradford protein quantification kit according to the manufacturer’s instructions. Raw mass spectrometry (MS) data were acquired through liquid chromatography-tandem mass spectrometry (LC-MS/MS) analysis in data-independent acquisition (DIA) mode. The resulting spectra from each fraction were separately searched against the Clusters of Orthologous Groups, Gene Ontology (GO), and Kyoto Encyclopedia of Genes and Genomes (KEGG) databases using the Proteome Discoverer 2.2 (PD 2.2, Thermo) search engine. Spectrogram data were converted into protein information, and data-dependent acquisition (DDA) quality control was performed simultaneously. The quality control data were imported into Spectronaut to construct a spectral library (DDA library). Subsequently, Spectronaut was used to compare the raw DIA scan files against the DDA library for protein identification, ultimately yielding 6657 proteins.

Metabolites were extracted using methanol, and the mixture was processed using a high-throughput tissue crusher, followed by vortexing and centrifugation. The supernatant was subjected to LC-MS/MS analysis. Chromatographic separation of the metabolites was carried out on a Thermo UHPLC system equipped with an ACQUITY BEH C18 column. Mass spectrometric data were collected using a Thermo UHPLC-Q Exactive Mass Spectrometer with an electrospray ionization source operating in both positive and negative ion modes. Data acquisition was performed in DDA mode. Following UPLC-TOF/MS analyses, the raw data were imported into Progenesis QI 2.3 (Nonlinear Dynamics, Waters, USA) for peak detection and alignment. Mass spectra of the metabolic features were identified by matching accurate mass, MS/MS fragment spectra, and isotope ratio differences against reliable biochemical databases, including the Human Metabolome Database (HMDB) (http://www.hmdb.ca/) and the Metlin Database (https://metlin.scripps.edu/). A total of 1165 metabolites were identified using the DDA method.

### Integrated analysis

Correlations between imaging features and RNAs, proteins, metabolites, pathways, and clinical factors were calculated using Spearman correlation analysis implemented in the “psych” package. Significantly different molecules across various clinical phenotypes were identified using the “DESeq2” package for RNA data, the “limma” package for protein data, and the Wilcoxon test for metabolite data and imaging feature. The association of molecules with overall survival was evaluated using Kaplan–Meier (KM) analysis.

### The imaging features predicting the gene expression level

Subsequently, 1691 genes were further selected from the initial 2297 genes in 141 patients based on a median TPM value > 1. Gene expression levels were classified into high and low expression groups using the median TPM value as the cutoff, which was predicted by imaging features. Three analysis pipelines were constructed following the 10 events per variable rule, based on the number of correlated imaging features: (i) genes with only one correlated imaging feature, (ii) genes with 2–14 correlated imaging features, and (iii) genes with more than 14 correlated imaging features. For genes with only one correlated imaging feature, that single feature was used to predict gene expression levels. For genes with 2–14 correlated imaging features, both the single imaging feature and a model developed using all correlated imaging features via logistic regression (LR) were employed for prediction. For genes with more than 14 correlated imaging features, predictions were made using the single imaging feature, a model developed with the top 14 correlated imaging features via LR, and a model developed using the least absolute shrinkage and selection operator (LASSO) combined with a 10-fold cross-validation (Figure S1).

Classical machine learning methods, including LR, random forest (RF), support vector machine (SVM), and LASSO, were employed to predict gene expression levels. Among these, RF exhibited a tendency toward overfitting, while SVM generally yielded lower Area under curve (AUC) values. In contrast, LASSO and LR demonstrated more stable performance (Figure S2). Consequently, we selected LASSO and logistic regression using the ‘glmnet’ package to develop the imaging-based gene expression prediction model.

### Database implementation

The processed data were imported into a MySQL database. The front-end dynamic display was developed using HTML/CSS in combination with the JavaScript library jQuery. The web interface interacts with the server through PHP 8.0 and MySQL, enabling seamless access and search functionality across both desktop and mobile devices.

## Results

### Overview of MIPD

**Figure 1.**
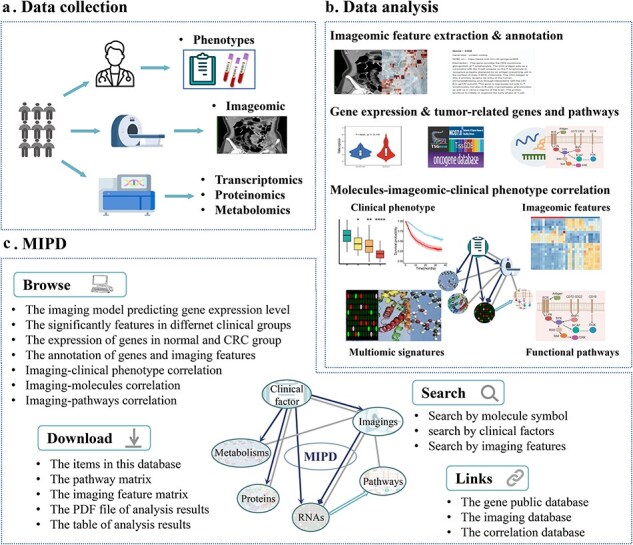
Database content and construction of MIPD. (a) Data collection process in the MIPD. (b) Construction of MIPD. (c) User interface of the MIPD, supporting browsing, searching, and downloading of analysis results.

**Figure 2.**
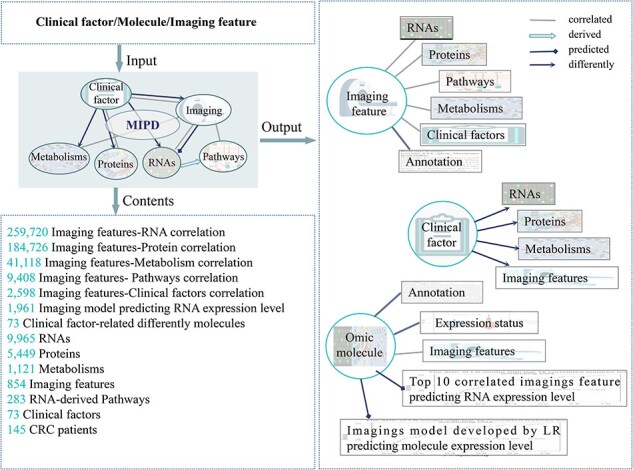
The main functions and usages of MIPD. Input, output, and contents information of MIPD.

We introduce the MIPD database, which provided a user-friendly, interactive, and big data interface, an integrated platform that elucidates the associations among molecules (RNAs, proteins, metabolites), imaging features, and clinical phenotypes. MIPD provides a user-friendly, interactive, and scalable big data interface. The workflow for data processing and an overview of MIPD are presented in Fig. 1. This database includes 591 CRC patients with imaging features, 142 with RNA-seq data, 31 with protein data, 56 with metabolomics data, and 159 with clinical data. Among these, 31 patients have complete multi-modal data, encompassing imaging, RNA-seq, protein, metabolite, and clinical information. The platform supports querying by molecular symbols, clinical factors, and imaging features (Fig. 2). Users can explore various data types, including correlations between molecules and imaging features, annotations of molecule and imaging features, expression and abundance profiles of RNA, proteins, and metabolites, as well as imaging features across distinct clinical groups. Additionally, the platform provides access to imaging-based models predicting gene expression levels. Data and results can be downloaded in multiple formats, including pathway matrices, imaging feature matrices, PDF files of analytical results, and tabulated summaries. External links to publicly available gene databases, imaging databases, and correlation databases are also integrated for further reference. In this study, we analyzed a comprehensive dataset comprising 497 570 correlations between imaging features, molecules, and clinical factors, 1961 imaging-based models predicting RNA expression levels, and 73 molecules associated with clinical factors (Fig. 2).

The MIPD platform comprises five main functional modules: “Home,” “Dataset,” “Search,” “Tutorial,” and “Contact.” The “Home” page provides a concise overview of the database and its objectives. The “Dataset” page provides the raw data and what we can search in this website. The “Search” page features a query interface allowing users to retrieve annotation information and analytical results by entering clinical factors, molecules, or imaging features. Additionally, a list of frequently queried genes is displayed below the search box for quick access to relevant results. The “Tutorial” page offers a step-by-step guide and methodological instructions to facilitate navigation and utilization of the platform. The “Contact” page includes the owner’s address and email for further communication. Users are encouraged to reach out for inquiries or to request access to the original data.

### Datasets

Additionally, users can download comprehensive tables containing gene symbols, proteins, metabolites, pathways, imaging features, and clinical factors that are searchable within the database. The Dataset page provides access to two key resources: a matrix of 283 pathway scores and an imaging feature matrix. Furthermore, external links to relevant databases, including TCGA, TCIA, GEO, and STRING, are conveniently located at the bottom of the page. These links enable users to access additional information on genes, gene expression profiles, CT imaging data, and protein–protein interactions.

### Molecule expression distribution

MIPD provides easy access to molecular information (RNA, protein, metabolite) based on gene symbols. The search page displays violin plots illustrating the distribution of molecular data in normal and tumor tissues, with *P*-values calculated using the *t*-test.

### Comparison with other databases

Currently, several databases, such as TCGA, TCIA, GEPIA, and starBase, provide access to gene metadata, gene expression profiles, and gene interaction networks. In contrast, MIPD distinguishes itself by offering unique resources, including imaging feature matrices, pathway matrices, and comprehensive correlations between imaging features and genes, proteins, metabolites, pathways, and clinical factors. Additionally, MIPD provides clinical factor-related molecules, pathways, and imaging features, along with KM analyses of significantly different features. Furthermore, the database includes ROC curves for imaging-based models predicting gene expression levels (Table 1).

**Table 1.** Comparison with the related databases

	TCGA	TCIA	GEPIA	starBase	MIPD
NA-seq data	√				√
Protein data	√				√
Metabolism data					√
Pathway data					√
CT data		√			
Imaging features					√
Gene expression			√	√	√
Gene–gene correlation			√	√	
Gene–protein correlation				√	
Gene-imaging feature correlation					√
Protein-imaging feature correlation					√
Metabolism-imaging feature correlation					√
Pathway-imaging feature correlation					√
ROC curve predicting the genes expression level by imaging features					√
Clinical factor-related molecules, pathways and imaging features					√

Abbreviation: TCGA: The Cancer Genome Atlas, TCIA: The Cancer Imaging Archive, GEPIA: Gene Expression Profiling Interactive Analysis, starBase: sRNA target Base, MIPD: Correlation of Genes and Imaging Features, CT: computer tomography, ROC: receiver operating characteristic curve.

### An example search

**Figure 3.**
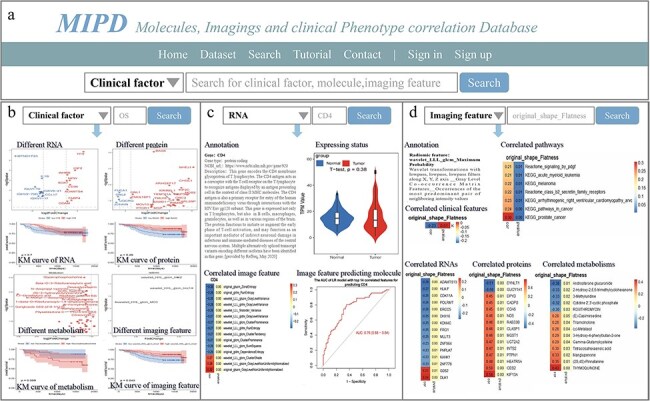
Overview of MIPD knowledgebase. (a) Homepage and keyword search interface. (b) Example of searching for the clinical factor ‘OS’. (c) Example of searching for the molecule ‘CD4’. (d) Example of searching for the imaging feature ‘original_shape_Flatness’.

The “Search” page enables users to perform basic database queries for clinical factors, molecules, and imaging features (Fig. 3a). For instance, by entering “OS” into the search box after selecting the “Clinical factor” option and clicking the search button (accessible from either the Home or Search page), the system displays volcano plots highlighting significantly different RNAs, proteins, metabolites, and imaging features, along with KM curves for the top significantly different features. A download link for the table of significantly different features is also provided on this page (Fig. 3b).

Similarly, when searching for RNA (e.g. CD4) by selecting the “RNA” option and clicking the search button, the page displays the gene type, NCBI URL, and gene description. Additionally, it presents a violin plot comparing gene TPM expression in normal versus cancer tissues, a heatmap showing correlations between the gene and imaging features, and ROC curves for single imaging features or LR models predicting gene expression levels. Download links for correlation and ROC results in both PDF and table formats are also available (Fig. 3c). Detailed analytical results, including tables of all gene-imaging feature correlations, model development data, AUC values for LASSO models, and the top 14 correlated single features, as well as PDF files containing gene expression levels in tumor and normal tissues, correlation heatmaps, and ROC curves for LR models, LASSO models, and the top 14 correlated single features, are provided in Figure S3.

When searching for an imaging feature (e.g. original_shape_Flatness, which exhibits significant correlations with other features), the page displays annotations for the imaging feature and heatmaps illustrating its correlations with clinical features, pathways, RNAs, proteins, and metabolites (Fig. 3d). Download links for correlation results in PDF and table formats are also provided. Detailed analytical results, including tables and PDF files summarizing all correlations between imaging features and RNAs, proteins, metabolites, pathways, and clinical factors, are available in the Supplementary Materials.

## Discussion

Currently, existing databases provide clinical phenotypes datasets, multi-omics datasets, and imaging datasets. However, a comprehensive understanding of the integrated relationships between molecules, imaging features, and phenotypes at different levels can significantly enhance clinicians’ ability to make informed decisions regarding diagnosis and treatment. To the best of our knowledge, MIPD is the first database to offer the following unique features: (i) correlations between molecules (RNAs, proteins, metabolites), pathways, clinical factors, and imaging features; (ii) prediction of gene expression levels based on imaging features; (iii) imaging models predicting RNA expression levels and identification of significantly different features across clinical factors; (iv) KM analyses of significantly different features stratified by clinical factors; and (v) imaging data, pathway data, and analysis results processed through a unified pipeline, all of which are available for download in both PDF and table formats.

In this study, we primarily focused on developing a database dedicated to multi-omics correlation analysis, with particular emphasis on exploring the associations between imaging features and other factors. The primary objective of this study is to provide robust multi-omics insights that enhance the application of imaging in disease diagnosis and treatment. Consequently, we prioritized the analysis of correlations between imaging features and other factors. Nevertheless, the investigation of relationships among RNAs, proteins, metabolites, and clinical factors also holds significant value and warrants further exploration. In the future, we plan to enhance our database through the following directions: (i) Integration of additional multi-omics data, such as genome and exome data, to explore their correlations with imaging features and clinical phenotypes. (ii) Incorporation of intercorrelation analyses among genes, RNAs, proteins, metabolites, and clinical factors. (iii) Development of correlation analyses between gene modules and imaging features. (iv) Implementation of an online analysis function that allows users to input genes and imaging features of interest for real-time analysis. (v) Expansion of visualization options for analytical results to improve data interpretation and user experience.

## Supplementary Material

baaf029_Supp

## Data Availability

The web interface of the database is available at http://corgenerf.com/. This website is freely accessible to all users. Please sign up and in if you want to download the analysis results. The code for this study is available in the https://github.com/jiaojiaozh/MIPD. The transcriptomic data can be found here: https://bigd.big.ac.cn/gsa-human/browse/HRA000235.
