# Supplementary material for: MIPD: Molecules, Imagings, and Clinical Phenotype Integrated Database
Source: Database (Oxford). 2025 Apr 21;2025:baaf029. doi: 10.1093/database/baaf029 (PMC12010968; doi:10.1093/database/baaf029)

Figure S2. The Receiver Operating Characteristic (ROC) curves of models developed by Least Absolute Shrinkage and Selection Operator (LASSO), Random Forest (RF), Support Vector Machine (SVM) and Logistic Regression (LR) methods for predicting the expression of ABL1 (A), ABL2 (B), ACTG2 (C), ACY1 (D), ADAM9 (E), ADAMTS8 (F), ADPRH (G). The genes shown are the first seven selected in alphabetical order.

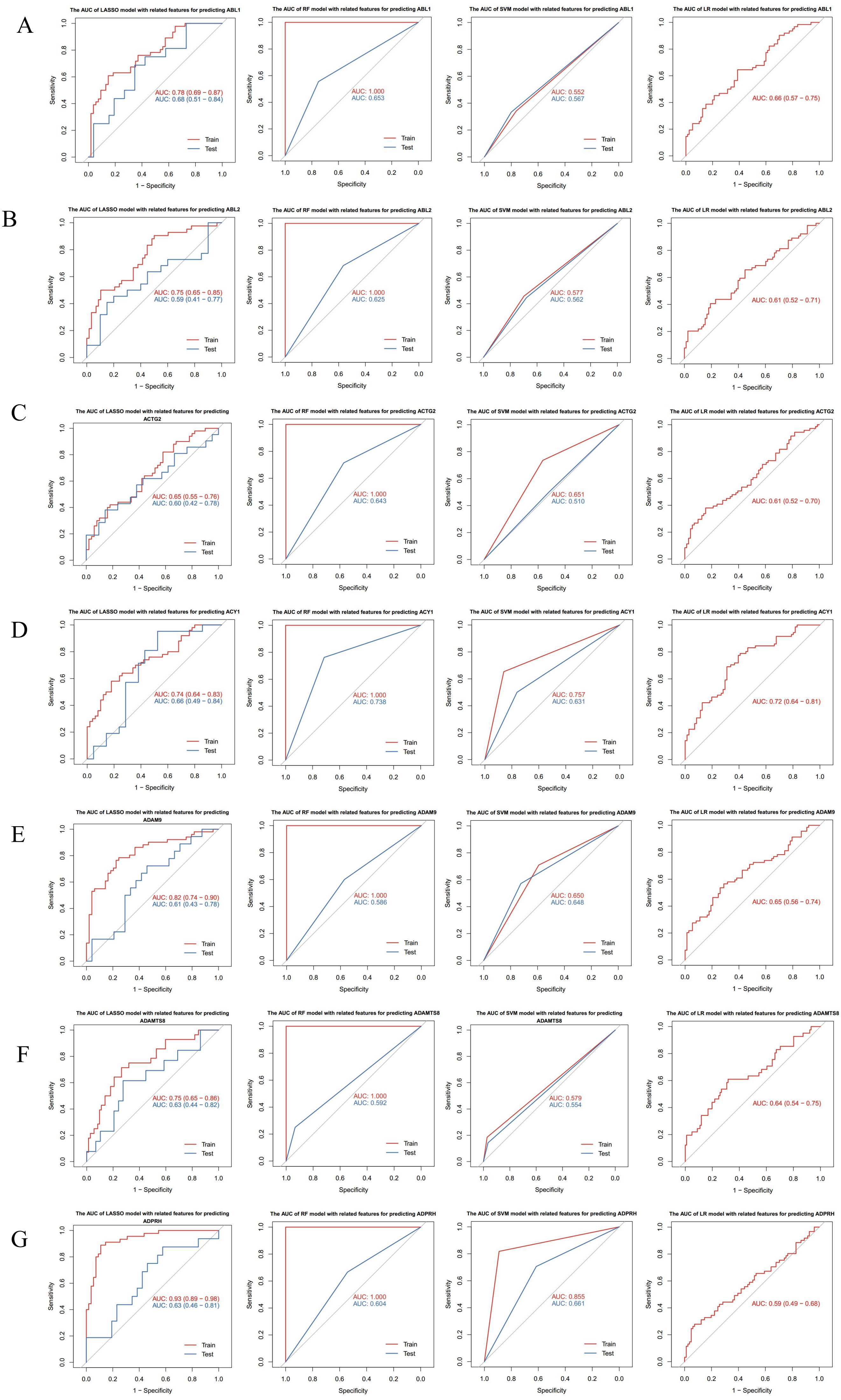

Supplement: baaf029_Supp [file baaf029_supp.zip › suppl_data/Figure S2.pdf]
