# Supplementary material for: MIPD: Molecules, Imagings, and Clinical Phenotype Integrated Database
Source: Database (Oxford). 2025 Apr 21;2025:baaf029. doi: 10.1093/database/baaf029 (PMC12010968; doi:10.1093/database/baaf029)

Figure S3. The analysis results of CD4 in this study. (A) The expression distribution of CD 4 in the normal and tumor group. (B) The correlation heatmap of CD4 with imaging features. Receiver Operating Characteristic (ROC) curves of imaging-based predictive models for CD4 expression levels, developed using logistic regression (C) and least absolute shrinkage and selection operator regression (D). ROC curves of the top 14 correlated-imaging featuresfor CD4 expression levels (E-R).

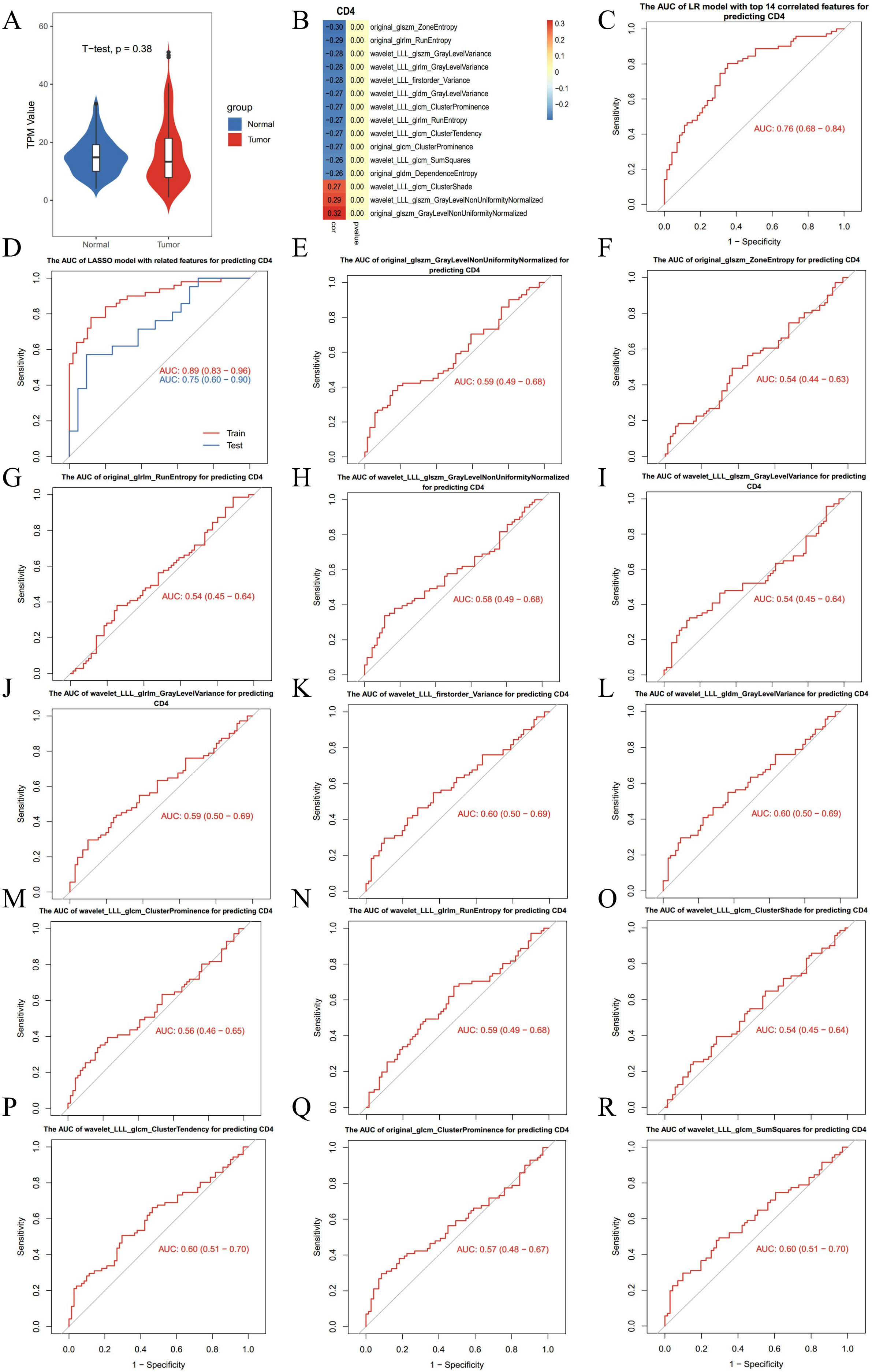

Supplement: baaf029_Supp [file baaf029_supp.zip › suppl_data/Figure S3.pdf]
