# Supplementary figures and images for: MIPD: Molecules, Imagings, and Clinical Phenotype Integrated Database
Source: Database (Oxford). 2025 Apr 21;2025:baaf029. doi: 10.1093/database/baaf029 (PMC12010968; doi:10.1093/database/baaf029)

Figure S1. Workflow of data processed.

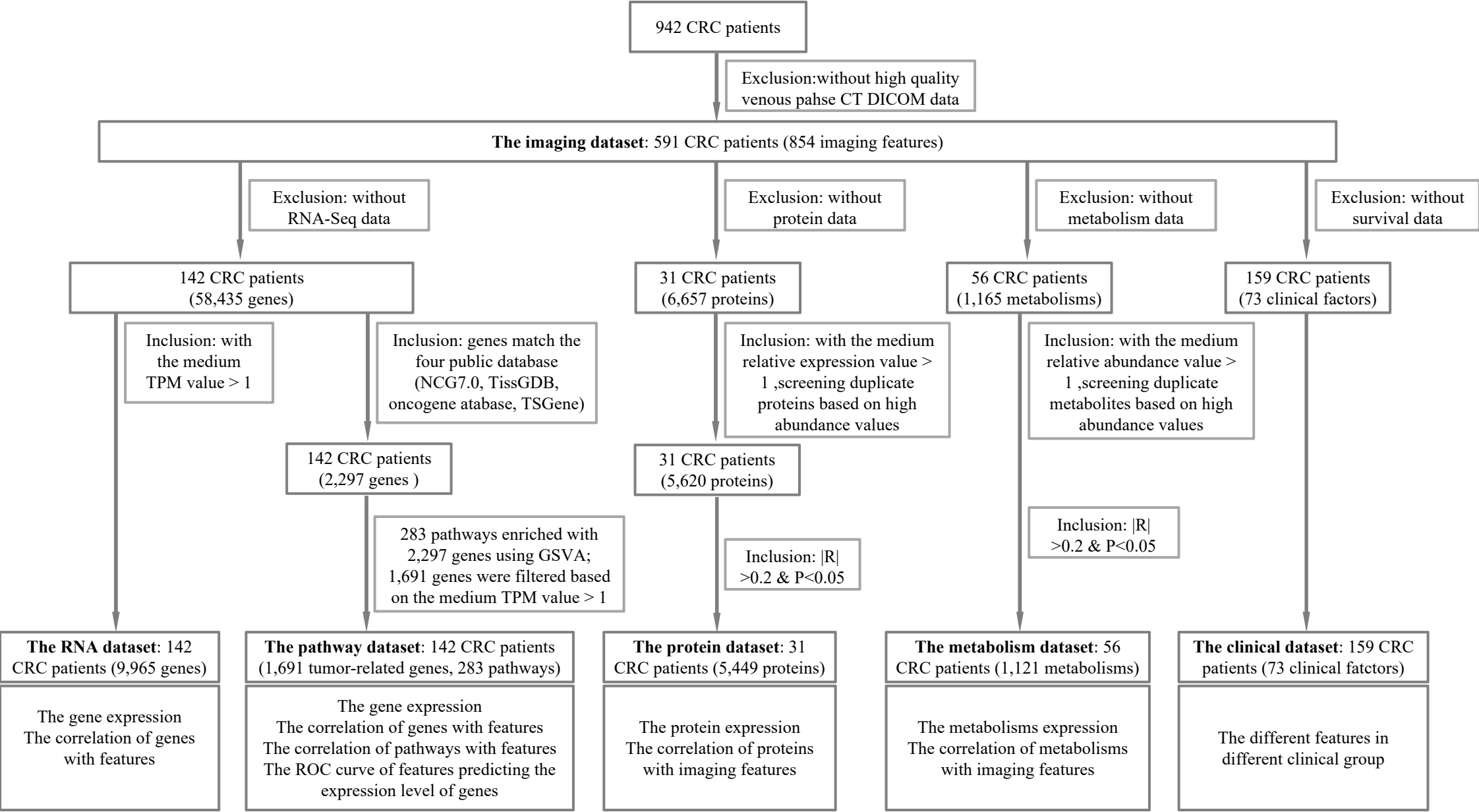

Supplement: baaf029_Supp [file baaf029_supp.zip › suppl_data/Figure S1.pdf]
